# Effects of miR-98 in intrauterine extracellular vesicles on maternal immune regulation during the peri-implantation period in cattle

**DOI:** 10.1038/s41598-019-56879-w

**Published:** 2019-12-30

**Authors:** Keigo Nakamura, Kazuya Kusama, Atsushi Ideta, Koji Kimura, Masatoshi Hori, Kazuhiko Imakawa

**Affiliations:** 1 Laboratory of Veterinary Pharmacology, Graduate School of Agricultural and Life Sciences, The University of Tokyo, Tokyo, 113-8657 Japan; 2 Department of Endocrine Pharmacology, Tokyo University of Pharmacy and Life Sciences, Tokyo, 192-0392 Japan; 3 Zen-noh Embryo Transfer Center, Fukuoka, 810-0001 Japan; 4 Graduate School of Environmental and Life Science, Okayama University, Okayama, 700-8530 Japan; 5 Research Institute of Agriculture, Tokai University, Kumamoto, 862-8652 Japan

**Keywords:** Developmental biology, Molecular biology

## Abstract

Evidence accumulated suggests that extracellular vesicles (EVs) present in uterine lumen play a role in conceptus-endometrial cell interactions during peri-implantation periods. However, how intrauterine EVs function on endometrium have not been well characterized. To study how intrauterine EVs affect endometrial milieu in cattle, bovine endometrial epithelial cells (EECs) were treated with EVs isolated from uterine flushing fluids (UFs) on day 17 or 20 pregnancy (P17, P20, respectively; conceptus implantation to endometrium begins on days 19–19.5). RNA extracted from EECs were then subjected to RNA sequence analysis. The analysis revealed that transcripts related to immune system were down-regulated in EECs treated with EVs on P20 compared with those on P17. To investigate whether microRNAs (miRNAs) in EVs regulate maternal immune system in the endometrium during the peri-implantation, microRNA sequence and in silico analyses were performed, identifying bta-miR-98 in EVs as a potential miRNA to regulate maternal immune system. Furthermore, the treatment of EECs with bta-miR-98 negatively regulated several immune system-related genes, *CTSC, IL6*, *CASP4* and *IKBKE*, in EECs. These results suggest that EVs containing bta-miR-98 is a regulator of maternal immune system, possibly allowing the conceptus attachment to the endometrial epithelium during the peri-implantation period.

## Introduction

Pregnancy is a unique physiological process and a complicated interaction between the conceptus and uterine endometrium is required for its establishment^1^. During the implantation period from blastocyst hatching to conceptus implantation to the endometrium in the bovine species, approximately 50% of pregnancy losses occur due possibly to insufficient communication between the elongating blastocyst and the maternal endometrium^2^. Although a large number of studies to identify transcripts present in conceptus or endometrium during early pregnancy have been conducted^3–11^, factors that regulate conceptus-endometrial communication or conceptus implantation have not been well characterized.

The communication between different cell types is generally considered to be the function of secretory soluble factors such as hormones and cytokines. In recent years, however, the role for exosomes and microvesicles, collectively termed extracellular vesicles (EVs), in cell-cell communication has been well-characterized. EVs contain lipids, proteins and nucleic acids, particularly microRNAs (miRNAs), which interact with specific target cells^12^. Evidence has been accumulated that EVs are present in most of bodily fluids as well as uterine flushing fluids (UFs)^13–16^. In ruminants, it has been reported that EVs isolated from uterine fluid, containing secretory products of conceptuses and/or endometrium during the peri-implantation period are potentially involved in trophoblast development and its implantation to the maternal endometrium^17,18^. In addition, previous studies demonstrated that bodily fluid-derived miRNAs could play roles in the preparation of maternal tissues for implantation, and in feto-maternal signaling during embryo development^19,20^. However, the effects of intrauterine EVs containing numerous bio-molecules including miRNAs on conceptus implantation to the endometrium during implantation periods have not been well-characterized.

Our previous study has demonstrated that the bovine *in vitro* conceptus implantation to endometrium model using bovine trophoblast cells and endometrial epithelial cells (EECs)^21^ requires UFs on pregnant day 17 or 20 (P17 or P20; conceptus implantation to EECs begins on days 19–19.5) to mimic the gene expression in utero on day 17 or 20, respectively. These results suggest that UF components during the conceptus implantation period, including various cytokines and/or EVs, are essential for biochemical and/or physical interactions between the conceptus and the endometrium. Although several global analyses with bovine UFs from early pregnant cows have also demonstrated changes in intrauterine protein levels^22–24^, intrauterine factors that initiate and/or drive conceptus implantation have not yet been identified. Based on these findings, we hypothesized that EVs present in bovine UFs during conceptus implantation period could regulate the endometrial milieu, facilitating conceptus attachment to the uterine epithelium.

Using RNA-seq analysis in the present study, EVs extracted from UFs during pre- (P17) and post-implantation (P20) periods were characterized and transcript changes in cultured EECs treated with EVs were examined. In silico analysis was then used to reveal significantly enriched or decreased molecular functions in EECs and the potential function of miRNA in EVs on the changes of immune system/response in EECs was further investigated by the use of miRNA-seq analysis and real-time PCR analysis.

## Results

### EVs are present in bovine UFs on pregnant days 17 and 20

**Figure 1:**
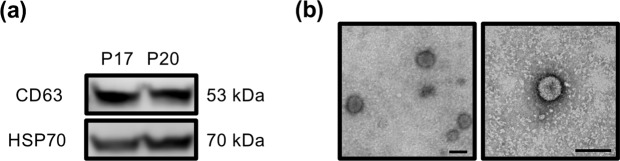
Characterization of EVs isolated from UFs during the peri-implantation period. (**a**) Western blot analysis showed the presence of CD63 and HSP70 in pellets isolated from P17 or P20 bovine UFs. Three independent experiments were done, and a representative one is shown. (**b**) Transmission electron microscopy analysis revealed the presence of 50–150 nm vesicles in UFs, consistent with those of EVs. Scale bar = 200 nm.

Using western blots, EVs in bovine UFs were characterized by the presence of EV markers, CD63 and HSP70, in the precipitates isolated from P17 and P20 UFs (Fig. 1a). Moreover, the transmission electron microscope (TEM) revealed the presence of 50–150 nm in diameter vesicles in the isolated EVs (Fig. 1b). These results indicated that EVs were secreted into the uterine lumen during peri-implantation periods.

### Transcriptome analysis of EECs treated with intrauterine EVs during peri-implantation period

**Figure 2:**
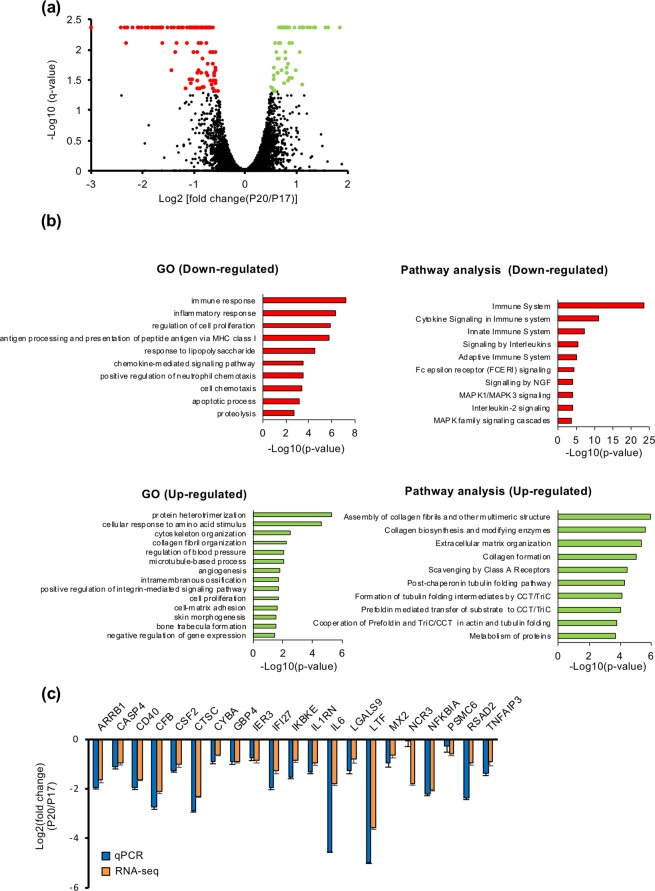
Transcript changes in bovine endometrial epithelial cells treated with intrauterine EVs during the peri-implantation period. (**a**) RNAs were extracted from EECs treated with EVs isolated from P17 and P20 UFs (n = 3 each). Volcano plot shows 179 differentially expressed genes identified by RNA-seq analysis, in which 67 genes had 2-fold up-regulation (green dots) and 112 genes showed 2-fold down-regulation (red dots) in EECs treated with EVs on P20 compared with those on P17. (**b**) Down- and Up-regulated transcripts in EECs were functionally classified by the biological process in enriched GO terms and enriched pathway analyses. (**c**) Levels of immune system-related transcripts in EECs treated with EVs on P17 and P20 (n = 3 each) were examined by qPCR or RNA-seq analysis. *ACTB* and *GAPDH* mRNA served as internal controls for RNA integrity.

To study effects of EVs from P17 and P20 UFs on gene expression in EECs, RNA sequencing (RNA-seq) analysis was performed, detecting 179 differentially expressed genes (DEGs) (Fig. 2a). Among 179 DEGs, 112 genes were down-regulated and 67 genes were up-regulated in EECs treated with EVs on P20 compared with those on P17 (Fig. 2a). Gene Ontology (GO) term and pathway most enriched by up-regulated genes were “protein heterotrimerization” and “assembly of collagen fibrils and other multimeric structures” (Fig. 2b). Regarding GO and enriched pathway analyses of the down-regulated genes, the most enriched were “immune response” and “immune system” (Fig. 2b), from which “immune system” in enriched pathway exhibited the lowest P-value. The details of these analyses are summarized in Tables 1 and 2. Therefore, we selected 21 transcripts, which were related to “immune system” in Table 2, for further analysis. Using qPCR, we ascertained the effect of P17 and P20 EVs on the expression of immune system-related genes in EECs. The results of *ARRB1, CASP4, CD40, CFB, CSF2, CTSC, CYBA, GBP4, IER3, IFI27, IKBKE, IL1RN, IL6, LGALS9, LTF, MX2, NFKBIA, PSMC6, RSAD2* and *TNFAIP3* are similar to those obtained from the RNA-seq analysis, whereas the expression of *NCR3* was different (Fig. 2c).

**Table 1:** Genes related to GO induced by EVs on P17 and P20 in EECs.

GO_BP Term	P-value	Genes
**Down-regulation**		
immune response	5.82E-08	CSF2, BOLA, CXCL5, CXCL3, CXCL2, CXCL8, CD40, TNFAIP3
inflammatory response	5.15E-07	NFKBIZ, CYBA, CASP4, CXCL5, PTGS2, CXCL3, CXCL2, CXCL8, CD40, PTGFR, ECM1
regulation of cell proliferation	1.27E-06	CDCA7, CXCL5, PTGS2, CXCL3, CXCL2, EGLN3, NFKBIA, CXCL8, CD40
antigen processing and presentation of peptide antigen via MHC class I	1.90E-06	BOLA
response to lipopolysaccharide	3.32E-05	CXCL5, CXCL3, CXCL2, NFKBIA, CXCL8, CD40
chemokine-mediated signaling pathway	3.02E-04	CXCL5, CXCL3, CXCL2, CXCL8
positive regulation of neutrophil chemotaxis	3.47E-04	CXCL3, CXCL2, CXCL8
cell chemotaxis	3.91E-04	CXCL5, CXCL3, CXCL2, SAA3
apoptotic process	7.77E-04	BCL2L15, CDCA7, CASP4, ARRB1, HMOX1, BCAP29, EGLN3, ZC3H12A
proteolysis	0.00186	PLAT, ECE1, CASP4, CFB, MMP9, LTF, MMP1
**Up-regulation**		
protein heterotrimerization	5.20E-06	COL6A2, COL1A2, COL6A1, COL1A1
cellular response to amino acid stimulus	2.91E-05	COL3A1, COL1A2, COL6A1, COL1A1, MMP2
cytoskeleton organization	0.00345	TUBB2B, FITM2, TUBB6, TUBB3
collagen fibril organization	0.0061	COL3A1, COL1A2, COL1A1
regulation of blood pressure	0.00832	ACTA2, PTGS1, COL1A2
microtubule-based process	0.00929	TUBB2B, TUBB6, TUBB3
angiogenesis	0.01698	CTGF, TGFBI, PLCD1, MMP2
positive regulation of integrin-mediated signaling pathway	0.02009	LIMS2, FLNA
intramembranous ossification	0.02009	COL1A1, MMP2
cell proliferation	0.0209	HRAS, TGFBI, FURIN, MIF

**Table 2:** Genes related to intrauterine EVs-induced enriched pathways in EECs.

Enrichment pathway	P-value	Genes
**Down-regulation**		
Immune System	4.01E-24	CD40, IL1RN, CSF2, RSAD2, MX2, TNFAIP3, CYBA, ARRB1, NFKBIA, IL6, NCR3, PSMC6, IFI27, CASP4, LGALS9, IKBKE, GBP4, CTSC, CFB, LTF, IER3
Cytokine Signaling in Immune system	6.19E-12	CD40, IL1RN, IL6, PSMC6, CSF2, RSAD2, IFI27, MX2, ARRB1, LGALS9, GBP4
Innate Immune System	5.15E-08	NFKBIA, PSMC6, CSF2, CASP4, TNFAIP3, ARRB1, IKBKE, CFB, IER3
Signaling by Interleukins	2.31E-06	IL1RN, IL6, PSMC6, CSF2, ARRB1, LGALS9
Adaptive Immune System	8.26E-06	NFKBIA, CD40, NCR3, PSMC6, CYBA, CTSC, IER3
Fc epsilon receptor (FCERI) signaling	4.59E-05	NFKBIA, PSMC6, CSF2, ARRB1, IER3
Signaling by NGF	8.52E-05	NFKBIA, PSMC6, CSF2, ARRB1, IER3
MAPK1/MAPK3 signaling	1.05E-04	IL6, PSMC6, CSF2, ARRB1
Interleukin-2 signaling	1.24E-04	PSMC6, CSF2, ARRB1, LGALS9
MAPK family signaling cascades	1.97E-04	IL6, PSMC6, CSF2, ARRB1
**Up-regulation**		
Assembly of collagen fibrils and other multimeric structures	1.01E-06	COL1A1, COL3A1, COL1A2, COL6A2, COL6A1
Collagen biosynthesis and modifying enzymes	2.19E-06	COL1A1, COL3A1, COL1A2, COL6A2, COL6A1
Extracellular matrix organization	4.61E-06	COL1A1, COL3A1, COL1A2, SPARC, MMP2, COL6A2, COL6A1, FURIN
Collagen formation	9.67E-06	COL1A1, COL3A1, COL1A2, COL6A2, COL6A1
Scavenging by Class A Receptors	3.35E-05	COL1A1, COL3A1, COL1A2
Post-chaperonin tubulin folding pathway	5.29E-05	TUBB2B, TUBB6, TUBB3
Formation of tubulin folding intermediates by CCT/TriC	7.84E-05	TUBB2B, TUBB6, TUBB3
Prefoldin mediated transfer of substrate to CCT/TriC	9.92E-05	TUBB2B, TUBB6, TUBB3
Cooperation of Prefoldin and TriC/CCT in actin and tubulin folding	1.66E-04	TUBB2B, TUBB6, TUBB3
Metabolism of proteins	2.09E-04	DYNC1H1, TUBB2B, RPS28, TUBB6, TUBB3, MMP2, UBC, MLEC, FURIN, TGFBI, ADAMTS12, RENBP

### Identification of potential miRNA, bta-miR-98, in EVs for the modification of maternal immune system

**Figure 3:**
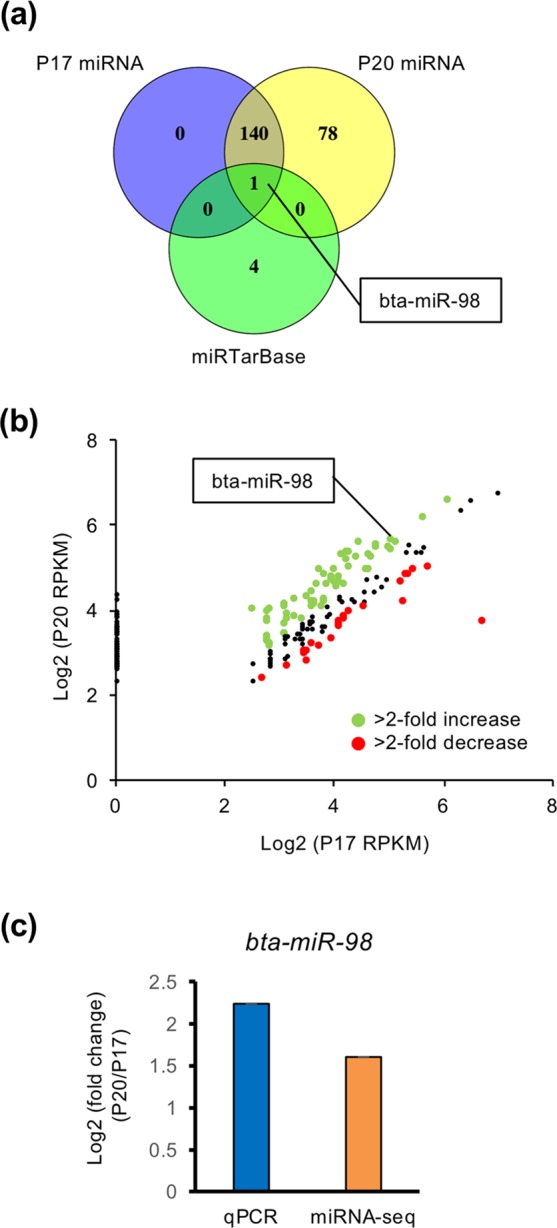
Bta-miR-98 in EVs was identified as a potential miRNA to require the modification of maternal immune system. (**a**) Venn diagram shows the number of miRNA-seq-identified miRNAs in P17 and P20 EVs, and that of miRTarBase-identified miRNAs related to immune system-related genes, among which only bta-miR-98 was overlapped. (**b**) Pair plot comparison of miRNAs on P20 with that on P17. (**c**) Fold changes in *bta-miR-98* in EVs on P20 compared with that on P 17 (n = 3 each) were examined by qPCR or miRNA-seq analysis. U6 served as an internal control for RNA integrity.

It has been reported that miRNAs in EVs participate in dynamic changes in uterine gene expression patterns^25^. To investigate whether miRNAs in EVs regulate maternal immune system in the endometrium during the peri-implantation period, identified immune system-related genes were then subjected to the analysis using the miRTarBase, database of experimentally validated miRNA-target interactions. Table 3 lists potential five miRNAs, miR-942, miR-146a, miR-98, miR-4670 and miR-6837, which are possible to down-regulate immune system-related genes in EECs treated with the P20 EVs. To further characterize functional miRNAs in P20 EVs, miRNA in EVs isolated from P17 and P20 UFs were subjected to miRNA-seq analysis, detecting 141 (within blue circle) and 219 (within yellow circle) miRNAs in P17 and P20 EVs, respectively (Fig. 3a). Among Venn diagram, potential miRNAs identified by miRTarBase were shown in green circle, in which a possible miRNA related to immune system-related genes was only bta-miR-98. Furthermore, the analysis found that 24 miRNAs exhibited 2-fold decrease and 61 miRNAs showed 2-fold increase, including bta-miR-98, in P20 EVs compared with those in P17 (Fig. 3b). In addition, qPCR analysis revealed that change in *bta-miR-98* was similar to that identified from the miRNA-seq analysis (Fig. 3c).

**Table 3:** Candidate miRNAs related to immune system.

miRTarBase	P-value	Genes
**Predicted miRNAs**		
miR-942	5.18E-05	NFKBIA, IFI27, GBP4, LTF
miR-146a	5.18E-05	IL6, RSAD2, IFI27, MX2
miR-98	0.00110773	IL6, ARRB1, CTSC, CFB, IER3
miR-4670	0.003891864	MX2, TNFAIP3
miR-6837	0.004603998	TNFAIP3, LTF

### Bta-miR-98 down-regulated immune system-related genes in EECs

**Figure 4:**
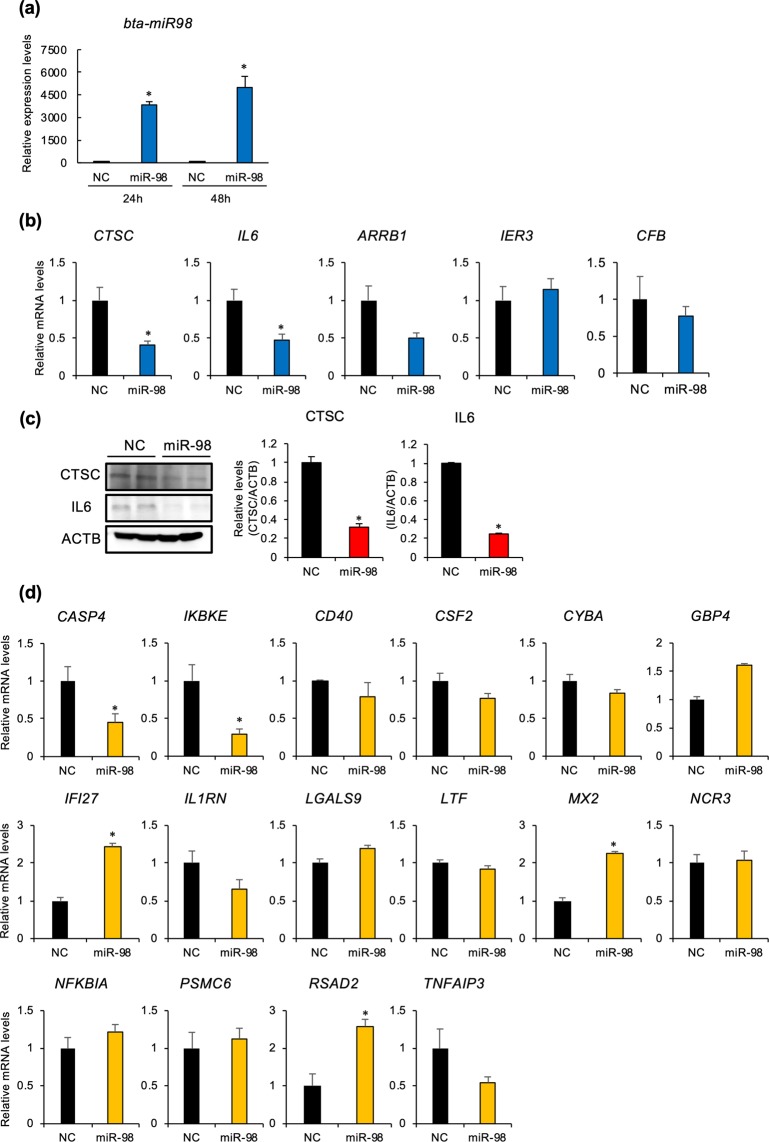
Effects of bta-miR-98 on the expression of immune system-related genes in EECs. EECs were transfected with or without bta-miR-98 for 24 or 48 h. (**a**) RNAs were extracted and subjected to qPCR to examine the uptake of miR-98 by EECs. U6 served as an internal control for RNA integrity. *p < 0.01 *vs*. negative control (NC). (b) RNAs were extracted and subjected to qPCR to examine potential target genes of miR-98. *ACTB* and *GAPDH* mRNAs served as internal controls for RNA integrity. *p < 0.05 *vs*. NC. (**c**) Cell lysates were subjected to western blot analysis. The bar graphs show CTSC or IL6 levels normalized by ACTB levels (n = 3 each). *p < 0.05 *vs*. NC. (**d**) RNAs were subjected to qPCR to examine immune system-related genes. *ACTB* and *GAPDH* mRNAs served as internal controls for RNA integrity. *p < 0.05 *vs*. NC. Data were from three independent *in vitro* culture experiments.

To explore the molecular mechanisms by which bta-miR-98 regulates maternal immune system, synthetic bta-miR-98 was added into the cultured EECs (Fig. 4a). Compared to the negative control, potential target genes of bta-miR-98 on immune system, *CTSC* and *IL6*, were down-regulated by bta-miR-98 (Fig. 4b). In addition, we examined the effect of bta-miR-98 on these protein expression, and found that CTSC and IL6 proteins were also down-regulated by bta-miR-98 (Fig. 4c). We further investigated the expression of down-regulated immune system-related genes in EECs treated with P20, and identified that *CASP4* and *IKBKE* were down-regulated by bta-miR-98 (Fig. 4d).

## Discussion

This study provides the evidence that miRNAs in EVs down-regulated the expression of maternal immune system-related genes. Pellets isolated from bovine P17 and P20 UFs were in fact EVs because of their size and morphology as seen by TEM and the presence of EVs protein markers. These observations are consistent with previous studies, in which EVs are present within the uterine lumen in cyclic as well as pregnant ruminants^26,27^. Global analysis of RNAs extracted from bovine EECs treated with intrauterine EVs revealed that P20 EVs up- and down-regulated endometrial gene expression related to assembly of collagen fibrils and other multimeric structures and immune system, respectively. Since the expressions of immune-related genes in EECs were most affected by the treatment with P20 EVs, immune system was selected for further analysis. The miRNA-seq and in silico analyses using the miRNA database identified bta-miR-98 in the EVs as one of potential miRNAs to regulate maternal immune system during peri-implantation period. This was also confirmed by the experiments with cultured bovine EECs with or without synthetic bta-miR98 treatment, in which synthetic bta-miR-98 down-regulated the four immune system-related genes out of twenty-one genes down-regulated by P20 EVs. These findings support the hypothesis that EVs’ components could regulate the uterine environment suitable for conceptus implantation to the uterine epithelium, and suggest that intrauterine bta-miR-98 in EVs could be one of the regulating maternal immune system in cooperation with the other components in EVs during the peri-implantation period.

Bioinformatics tools to predict miRNA-target and miRNA-seq identified bta-miR-98 in EVs, which down-regulated transcripts related to maternal immune system after the conceptus implantation. We further demonstrated that the bta-miR-98 transfection negatively regulated several immune-related genes such as CTSC, IL6, CASP4 and IKBKE in EECs. It was reported that miR-98 expression is associated with the induction of embryo implantation through controlling the uterine cell apoptosis during the pre-implantation period in rats^28^, and that miR-98 impairs the immune regulatory system in B cells^29^. Our previous study also demonstrated that the treatment of EECs with P20 EVs, not P17, down-regulated apoptosis-related genes^26^. These results suggest that bta-miR-98 found in P20 EVs could collaborate with other miRNAs and/or their components and regulate maternal immune system as well as apoptosis of endometrial epithelial cells, all of which are required for successful conceptus implantation.

EVs contain several nucleic acids, among which miRNAs are notable components, suggesting that EVs can serve as a means of interaction of one cell to another^20^. It has been shown that several miRNAs in the embryo- and/or endometrium-derived EVs including exosomes affect the expression of adhesion- and migration-related genes in the endometrium^30^. In addition, bioinformatics analysis of the EV-derived miRNAs identified their involvement in biochemical pathways necessary for embryo-endometrial cross talk at implantation, inflammation, cell remodeling, proliferation, and angiogenesis^31,32^. In this study, the miRNA profiles from bovine EVs revealed the presence of 219 miRNAs, of which 85 miRNAs were differently expressed in the P17 and P20 uteri, before and right after conceptus implantation is initiated, respectively. These results indicate that miRNA components, including bta-miR-98, in EVs could control gene expression in endometrium and conceptus, which could have paved uterine environments required for pregnancy establishment.

EVs, particularly exosomes, have been shown to regulate immune effects^33^. In mammals, maternal immunotolerance to the fetal allograft is essential when fetal development and growth proceed in the uterus. Earlier study reported that EVs contain the immunosuppressive HLA-G, which assists embryo development through the maternal immunotolerance^34^. In addition, it has been reported that lymphocytes are induced in response to pregnancy to protect conceptus from maternal immune system during the implantation period^35^. In that study, uterine immune cells in pregnant cows could be directly induced by IFNT. Our previous study revealed that ovine and bovine EVs contain IFNT, indicating that some of EVs present in utero^26,27^. Taken together, these findings suggest that EVs containing IFNT could act as one of the intermediators for the suppression of immune activation in uterine immune cells as well as the bovine endometrium during the conceptus implantation period.

In conclusion, we suggest that EVs containing bta-miR-98 released into the uterine lumen is an essential regulator of maternal immune system, allowing conceptus implantation to the uterine endometrium during the peri-implantation period.

## Materials and Methods

### Collection of bovine uterine flushing fluids

Donor Japanese black cows (3–7 years old) and recipient Holstein heifers (14–18 months old) were subjected to this study. All experimental and non-surgical procedures were performed in compliance with the guidelines for the Husbandry and Management of the Laboratory Animals of Zen-noh Embryo Transfer (ET) Center, and approved by the Zen-noh ET Center Animal Experiment Committee. The approval was also obtained from the Ethics Committee of the University of Tokyo (IRB number 7A-6–605). For ET processes, day 7 embryos (day 0 = day of estrus) were collected from super-ovulated and artificially inseminated Japanese black cattle. Two blastocysts were transferred non-surgically into the uterine horn, ipsilateral to the corpus luteum, of recipient cows on day 7 of the estrous cycle. UFs and elongated conceptuses on P17 or P20 (n = 3/day) were collected non-surgically by uterine flushing with 500 ml sterile phosphate-buffered saline (PBS, pH 7.2), as described previously^36^. Cell debris from the media were removed by centrifugation at 4000 x g for 5 min and the supernatant were filtered through 0.22 µm membrane and stored at −80 °C until use.

### Isolation of EVs from uterine flushing fluids

EVs from P17 and P20 UFs were isolated with the addition of exosome precipitation solution (Exo-Quick-TC, System Biosciences, Mountain View, CA, USA), which were incubated overnight at 4 °C according to the manufacturer’s instructions. The UFs with Exo-Quick-TC were then centrifuged at 1500 x g for 30 min at 4 °C. EVs were suspended and their protein concentration adjusted either in PBS (1 µg/µl) for TEM analysis and *in vitro* culture experimentations, whereas EVs were suspended in mammalian protein extraction reagent (M-PER, Thermo Fisher Scientific, Waltham, MA, USA) for western blot analysis^26,27^.

### Western blot analysis

EV marker and immuno-related proteins in lysates from EVs and EECs were detected through the use of SDS-PAGE. Separated proteins transferred onto polyvinylidene difluoride (PVDF) membranes (Bio-Rad, Hercules, CA, USA) were blocked with Block Ace reagent (DS Pharma Biomedical, Osaka, Japan), and the membranes were incubated with the following antibodies; rabbit polyclonal anti-human CD63 antibody (1:2000, EXOAB-CD63A-1, System Biosciences), rabbit polyclonal anti-human HSP70 antibody (1:2000, EXOAB- HSP70A-1, System Biosciences), rabbit polyclonal anti-bovine CTSC (1:2000, ab182904, abcam, Tokyo, Japan), rabbit polyclonal anti-pocine IL6 (1:2000, ab193853, abcam), or rabbit polyclonal anti-human ACTB (1:5000, ab1801, abcam). The membranes were then incubated with secondary antibody, horseradish peroxidase labeled goat anti-rabbit IgG (1:5000, Vector Laboratories, Burlingame, CA, USA), and immunoreactive bands were detected using enhanced chemiluminescence (EMD Millipore, Temecula, CA, USA). Signals were detected using C-DiGit Blot Scanner (LI-COR) and then band density was assessed with Image Studio DiGit software (version 5.2)^37^.

### Transmission electron microscopy

After the EVs in PBS, placed on carbon-film grid, were partially dried, the staining solution of 2% uranyl acetate in water was added to grids for 2 min and the excess liquid was blotted off with filter paper. The grids were dried overnight at room temperature. Grids were analyzed through the use of a HITACHI H-7600 Transmission Electron Microscope (TEM, Hitachi High- Technologies Corporation, Tokyo, Japan) at Hanaichi UltraStructure Research (Aichi, Japan)^26,27^.

### Cell preparation and culture conditions

Isolation and culture of EECs was carried out as previously described^38^. In brief, uteri of healthy Holstein cows were obtained from a local abattoir in accordance with protocols approved by the local Institutional Animal Care, Use and Ethics Committee at Okayama University, Okayama, Japan. Uteri of the early luteal phase (days 2–5) were excised and immediately transported to the laboratory. To detach EECs, the uterine lumen was trypsinized (0.3% w/v), from which EECs were isolated. EECs were then cultured on collagen type IA-coated plates in Dulbecco modified Eagle medium/F12 (DMEM/F12) (1:1) medium (Wako Pure Chemical Industries, Osaka, Japan) supplemented with 10% (v/ v) newborn calf serum (Thermo Fisher Scientific, Tokyo), 2 mM glutamine (Thermo Fisher Scientific), and antibiotic/antimycotic solution (Thermo Fisher Scientific) at 37 °C under 5% CO_2_ in humidified air. EECs (5 × 10^4^ cells/well) placed onto collagen type IA-coated 12-well plate were further incubated with P17 or P20 EVs (10 µg/well) in serum-free DMEM/F12 for 48 h. Cell viability was not altered with the use of serum-free medium in our culture system. For transfection of miRNA, EECs (5 × 10^4^ cells/well) cultured in 12-well plate were transfected with miRNA the synthetic bta-miR-98 (45 pmol/well, *mir*Vana miRNA Mimic, MC10426, Thermo Fisher Scientific) or negative control (45 pmol/well, *mir*Vana miRNA Mimic, 4464059, Thermo Fisher Scientific) for 48 h using the Lipofectamine RNAiMAX reagent (3 μl/well, Thermo Fisher Scientific) respectively.

### RNA extraction and quantitative RT-PCR

Total RNAs were extracted from cultured EECs or isolated EVs using the ISOGEN reagent (Nippon Gene, Tokyo, Japan) or SeraMir Exosome RNA Amplification Kit (System Biosciences), respectively, according to the manufacturer’s protocols. Reverse-transcription of the isolated RNAs was performed with ReverTra Ace qPCR RT Kit (Toyobo, Osaka, Japan), which was then subjected to qPCR amplification using PowerUP SYBR Green Master Mix (Thermo Fisher Scientific). For miRNAs analysis, the miRNA First-Strand Synthesis Kit (Clontech, Tokyo, Japan) was used to synthesize micro-cDNA, then subjected to qPCR by using the SYBR Green (Clontech). Primers are listed in Table S1. Calibration curves were used to examine amplification efficiencies of each target gene and the reference genes, glyceraldehyde-3-phosphate dehydrogenase (*GAPDH*), actin beta (*ACTB*), and *U6*, and were found to be comparable. Sequence Detection System software v2.3 (Applied Biosystems) was used to determine average threshold (Ct) values for each target^39,40^.

### RNA sequencing, data, gene ontology and pathway analyses

Total RNA for RNA-seq analysis was extracted from cultured EECs using Isogen (Nippon gene) according to the manufacturer’s instructions. High-throughput sequencing libraries were prepared using the TruSeq Stranded mRNA LT Sample Prep Kit (Illumina, San Diego, CA, USA) according to the manufacturer’s instructions, and the analysis was performed by Macrogen Japan (Kyoto, Japan). Primary sequencing data were deposited to the DDBJ (DNA Data Bank of Japan) Sequence Read Archive (https://www.ddbj.nig.ac.jp/dra/index-e.html) (accession numbers DRR174788 to DRR174793). Data analysis was performed as described previously^41^. Briefly, trimmed sequences were analyzed on the basis of the TopHat/Cufflinks pipeline based on the bovine genome (bosTau8) and reference annotations obtained from UCSC genome browser (https://genome.ucsc.edu). Differential and significant gene expression analysis was performed with the use of gene-level FPKM (fragments per kilo-base of gene locus summarized mRNA per million reads) expression levels. Genes were selected with the criteria of an absolute expression level >1 FPKM. GO and enriched signaling pathway analyses were performed with the Enrichr tool (http://amp.pharm.mssm.edu/Enrichr/).

### miRNA sequencing

For miRNA-seq analysis, RNA was extracted from P17 and P20 EVs using SeraMir Exosome RNA Amplification Kit (System Biosciences) according to the manufacturer’s instructions. miRNA libraries were established using TailorMix Gel-Free miRNA Sample Preparation Kit (SeqMatic, Fremont, CA, USA) according to the manufacturer’s instructions. RNA sequencing was performed on an Illumina Hiseq. 2500 platform by Macrogen Japan, and 50-bp, single-end reads were generated. Trimmed reads of 18–50 nt were mapped to a reference sequence by Bowtie. Mapped fragments were annotated with known miRNA database (miRBase22.1) using featureCounts. Annotated data was normalized using edgeR to the total reads of each individual sample as the standardized to reads per kilobase of exon per million (RPKM). The primary data was deposited to the DDBJ Sequence Read Archive (accession numbers DRR174798 and DRR174799).

### Statistical analysis

All experimental data from qPCR analyses represent the results obtained from three or more independent experiments each with triplicate assays. Data are expressed as the mean ± SEM. Significance was assessed using Dunnet comparisons test. A P-value < 0.05 was considered statistically significant. In RNA-seq analysis, FDR-adjusted p-value (q-value) < 0.05 was considered statistically significant.

## Supplementary information

Supplementary_Information.
