# Supplementary material for: Effects of miR-98 in intrauterine extracellular vesicles on maternal immune regulation during the peri-implantation period in cattle
Source: Sci Rep. 2019 Dec 30;9:20330. doi: 10.1038/s41598-019-56879-w (PMC6937239; doi:10.1038/s41598-019-56879-w)

**SUPPLEMENTARY INFORMATION**

**Effects of miR-98 in intrauterine extracellular vesicles on maternal immune regulation during the peri-implantation period in cattle.**

**Keigo Nakamura^1^, Kazuya Kusama^1,2*^, Atsushi Ideta^3^, Koji Kimura^4^, Masatoshi Hori^1^, Kazuhiko Imakawa^5^**

^1^Laboratory of Veterinary Pharmacology, Graduate School of Agricultural and Life Sciences, The University of Tokyo, Tokyo, 113-8657, Japan

^2^Department of Endocrine Pharmacology, Tokyo University of Pharmacy and Life Sciences, Tokyo, 192-0392, Japan

^3^Zen-noh Embryo Transfer Center, Fukuoka, 810-0001, Japan

^4^Graduate School of Environmental and Life Science, Okayama University, Okayama 700-8530, Japan.

^5^Research Institute of Agriculture, Tokai University, Kumamoto, 862-8652, Japan

^*^kusamak@toyaku.ac.jp

**Table S1. Primers for real-time PCR analyses.**

| **Name (Accession No.)** | **Sequence** | **Product length (bp)** |
| --- | --- | --- |
| ARRB1 (NM_174243) | F: 5'- ACGCGGGTGTTCAAGAAGG -3' | 126 |
|  | R: 5'- ATACTCCGGATCCACAAGAACC -3' |  |
| CASP4 (NM_176638) | F: 5'- TCCTTGGAATGAAGCAGCTG -3' | 108 |
|  | R: 5'- CACGTGCAGCAAATTTCCAC -3' |  |
| CD40 (NM_001105611) | F: 5'- CGGTAAAGGCGAATTCTTGTCC -3' | 139 |
|  | R: 5'- GCCTTCGACACATACACAAGTG -3' |  |
| CFB (NM_001040526) | F: 5'- AACGGCCATCTGTGATGATG -3' | 107 |
|  | R: 5'- AGTAGGTGACACGGTCTTCAAG -3' |  |
| CSF2 (NM_174027) | F: 5'- GAATGACACAGAAGTCGTCTCTG -3' | 120 |
|  | R: 5'- AAGGAGCCCATGAGACTAGTG -3' |  |
| CTSC (NM_001033617) | F: 5'- ATACCTCGGAGAACGTGAACG -3' | 128 |
|  | R: 5'- CAGTCCAAGACTTCTGAATGGC -3' |  |
| CYBA (NM_174034) | F: 5'- TCAGTTCACCCAGTGGTACC -3' | 120 |
|  | R: 5'- TTCTGTCCACACCTCTCCATG -3' |  |
| GBP4 (NM_001102261) | F: 5'- AAGATGAGTACCTGGAGAGTGC -3' | 136 |
|  | R: 5'- CTTGCAGGACGGTCAAAGAC -3' |  |
| IER3 (NM_001075202) | F: 5'- TCCCGAGATCTTCACCTTCG -3' | 149 |
|  | R: 5'- GGTTCGGTTCCTCGACTGG -3' |  |
| IFI27 (NM_001038050) | F: 5'- GAATCACTGCCTCCTCCTTG -3' | 145 |
|  | R: 5'- CCCACCAAGAGTTTGGATGA -3' |  |
| IKBKE (NM_001046345) | F: 5'- GGCAGATTACAGCACAGCTAAG -3' | 123 |
|  | R: 5'- AGCATCTCCACGAACCAGTG -3' |  |
| IL1RN (NM_174357) | F: 5'- TCCACGGTTACCTAATCTGTC -3' | 153 |
|  | R: 5'- CAGCAACTAATTGGTTATTCCTC -3' |  |
| IL6 (NM_173923) | F: 5'- ACCGAAGCTCTCATTAAGCG -3' | 105 |
|  | R: 5'- TTCTGCCAGTGTCTCCTTGC -3' |  |
| LGALS9 (NM_001015570) | F: 5'- AGGTACAGAGCTCAGAGTTCAG -3' | 136 |
|  | R: 5'- TTCTGGAAGCTGATGGAGGAC -3' |  |
| LTF (NM_180998) | F: 5'- GGAAGCAGATGCCCTGAACT -3' | 141 |
|  | R: 5′- AGGTACCCTTCCGTTGGTCT -3′ |  |
| MX2 (NM_173941) | F: 5'- CAGAGACGCCTCAGTCGAAG -3' | 113 |
|  | R: 5'- GAGACGTTTGCTGGTTTCCATG -3' |  |
| NCR3 (NM_001040524) | F: 5'- AGAGCTCCAACTCACGCTTATC -3' | 121 |
|  | R: 5'- TGGATTGGGATGGATTGGAAGG -3' |  |
| NFKBIA (NM_001045868) | F: 5'- TCCTGCACTTAGCCATCATCC -3' | 145 |
|  | R: 5'- TCTGGCTGGTTAGTGATCACAG -3' |  |
| PSMC6 (NM_001046240) | F: 5'- GGCAGATTGTGGGTGAAGTAC -3' | 136 |
|  | R: 5'- GCAACTCTTGTTCCTGGCTTC -3' |  |
| RSAD2 (NM_001045941) | F: 5'- GTGGTTCCAGAAGTACGGTGA -3' | 103 |
|  | R: 5'- CTTCTTTCCTTGACCACGGC -3' |  |
| TNFAIP3 (NM_001192170) | F: 5'- TTGCAACATCCTCAGAAGGC -3' | 101 |
|  | R: 5'- AAATCCCACCCACCTTCAGAG -3' |  |
| ACTB (NM_173979) | F: 5'- ATATTGCTGCGCTCGTGGTTG -3' | 148 |
|  | R: 5'- TAGGAGTCCTTCTGGCCCATG -3' |  |
| GAPDH (NM_001034034) | F: 5'- GCATCCCTGAGACAAGATGGTG -3' | 113 |
|  | R: 5'- CATTGATGGCAACGATGTCCAC -3' |  |
| bta-miR-98 (NR_031360) | F: 5'- TGAGGTAGTAAGTTGTATTGTT -3' |  |

F: Forward, R: Reverse.

**Figure S1. Full images of western blots shown in Figure 1 and Figure 4.**

Red dotted lines indicate the cropping locations.
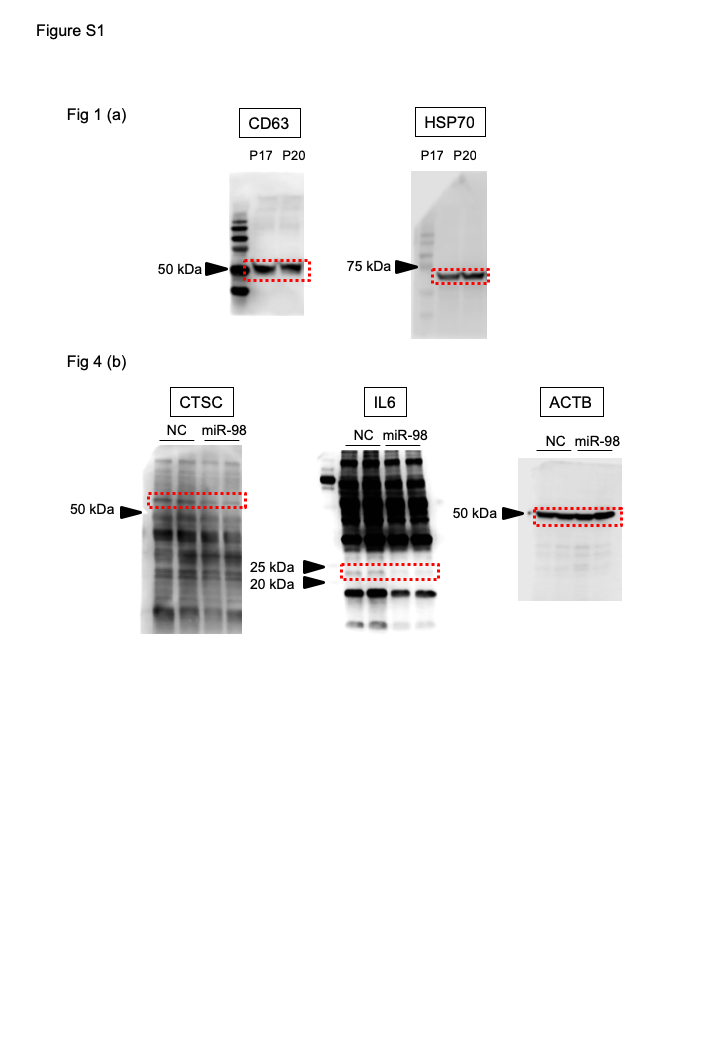

Supplement: Supplementary file 1 — Supplementary_Information. [file 41598_2019_56879_MOESM1_ESM.docx]
